# Boerhaave syndrome due to hypopharyngeal stenosis associated with chemoradiotherapy for hypopharyngeal cancer: a case report

**DOI:** 10.1186/s40792-018-0462-z

**Published:** 2018-06-08

**Authors:** Hideharu Tanaka, Norihisa Uemura, Daisuke Nishikawa, Keisuke Oguri, Tetsuya Abe, Eiji Higaki, Takahiro Hosoi, Byonggu An, Yasuhisa Hasegawa, Yasuhiro Shimizu

**Affiliations:** 1 Department of Gastroenterological Surgery, Aichi Cancer Center Hospital, 1-1 Kanokoden, Chikusa-ku, Nagoya, Aichi 464-8681 Japan; 2 Department of Head and Neck Surgery, Aichi Cancer Center Hospital, 1-1 Kanokoden, Chikusa-ku, Nagoya, Aichi 464-8681 Japan

**Keywords:** Boerhaave syndrome, Spontaneous esophageal rupture, Esophageal perforation, Chemoradiotherapy, Hypopharyngeal cancer

## Abstract

**Background:**

Spontaneous esophageal rupture, also known as Boerhaave syndrome, is a very serious life-threatening benign disease of the gastrointestinal tract. It is typically caused by vomiting after heavy eating and drinking. However, in our patient, because of a combination of hypopharyngeal cancer with stenosis and chemoradiotherapy (CRT), which caused chemotherapy-induced vomiting, radiotherapy-induced edema, relaxation failure, and delayed reflexes; resistance to the release of increased pressure due to vomiting was exacerbated, thus leading to Boerhaave syndrome. To the best of our knowledge, this is the first report of a patient with esophageal rupture occurring during CRT for hypopharyngeal cancer with stenosis.

**Case presentation:**

A 66-year-old man with a sore throat was referred to our hospital. He was found to have stage IVA hypopharyngeal cancer, cT2N2bM0, and underwent radical concurrent CRT consisting of weekly cisplatin (30 mg/m^2^) and radiation (70 Gy/35fr), for larynx preservation. On day 27 of treatment, he vomited, which was followed by severe left chest pain radiating to the back and the upper abdomen. Enhanced computed tomography (CT) revealed extensive mediastinal emphysema and a small amount of left pleural effusion. Esophagography revealed extravasation into the left thoracic cavity, and the patient was diagnosed with an intrathoracic rupture type of Boerhaave syndrome. He underwent emergency left thoracotomy 21 h after the onset. The ruptured esophageal wall was primarily repaired by closure with two-layer suturing and covered by a pedicled omentum. A jejunostomy tube was placed for postoperative enteral nutrition. On postoperative day (POD) 16, the patient was transferred to head and neck surgery to finish CRT and was discharged on POD 56. He has survived without relapse for 11 months after surgery.

**Conclusion:**

Patients with head and neck cancer are at risk for developing Boerhaave syndrome during CRT. In addition, since such patients often are in poor overall condition because of immunosuppression and protracted wound healing, Boerhaave syndrome can rapidly lead to severe life-threatening infections such as empyema and mediastinitis. Therefore, awareness of this condition is important so that appropriate treatment can rapidly be implemented to increase the likelihood of a good outcome.

## Background

Spontaneous esophageal rupture leading to full-thickness transmural rupture of a normal esophagus, also known as Boerhaave syndrome, is an emergency. Although it is typically caused by vomiting after heavy eating and drinking [1], Boerhaave syndrome can be caused by chemotherapy-induced vomiting. Furthermore, as in the case of advanced hypopharyngeal cancer with stenosis involving the oropharynx, vomiting-induced elevated pressure might not be relieved through the oropharynx, which therefore results in rapidly increased intraesophageal pressure. The recent organ-preserving treatment for pharyngeal cancer is aggressive concurrent chemoradiotherapy (CRT), which has obtained high rates of organ preservation [2]. However, some patients are adversely affected by the side effects of chemoradiotherapy, which include mucositis, dysphagia, and nausea and vomiting [3]. Here, we report a patient with hypopharyngeal cancer with stenosis, who developed esophageal rupture during CRT.

## Case presentation

**Fig. 1:**
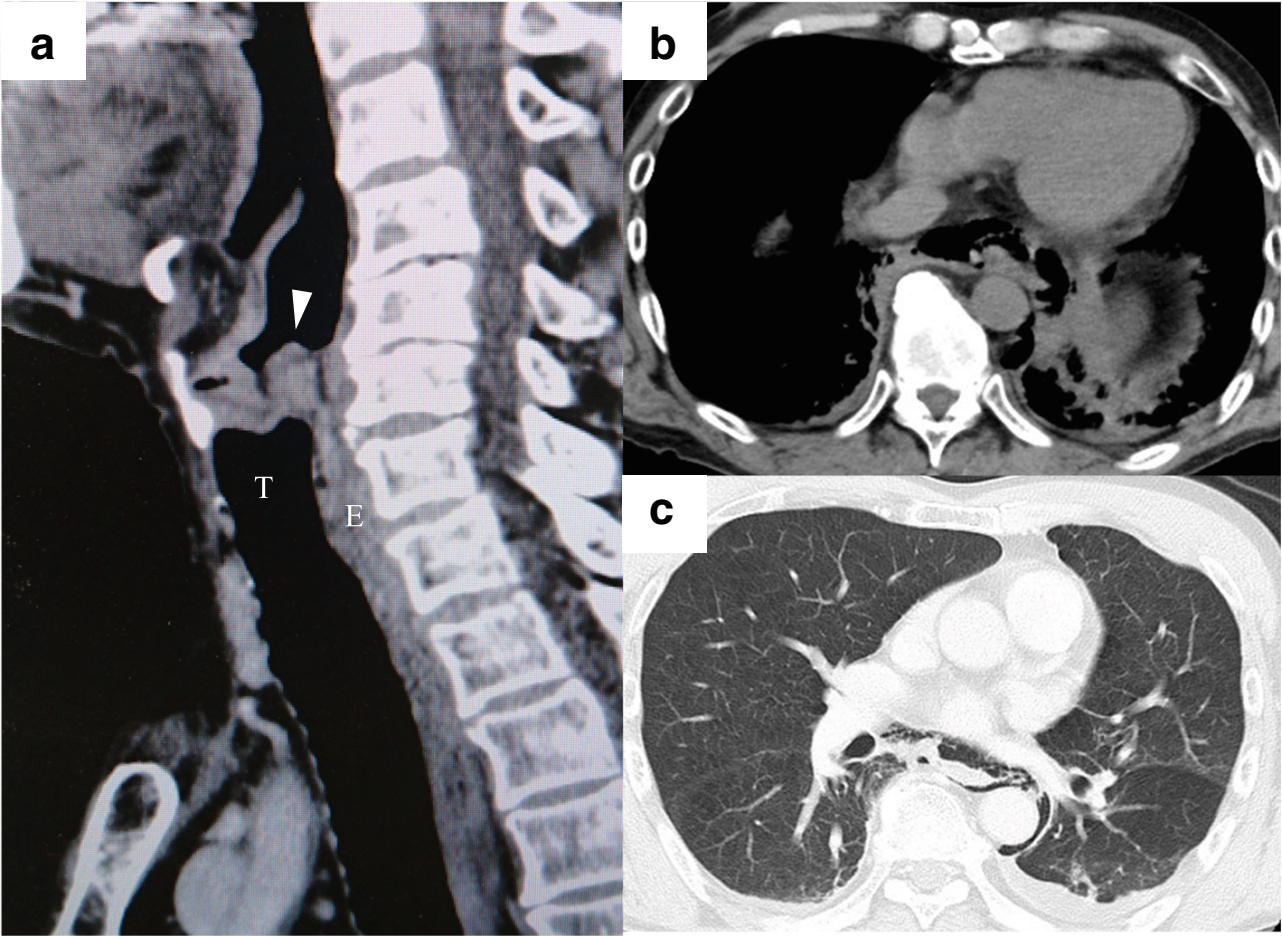
**a** Enhanced CT on sagittal section demonstrates stenosis caused by hypopharyngeal cancer involving the oropharnx (arrowhead, hypopharyngeal cancer). **b**, **c** Enhanced CT demonstrates extensive mediastinal emphysema, which ranged from the neck to the gastric cardia, and a small amount of left pleural effusion

**Fig. 2:**
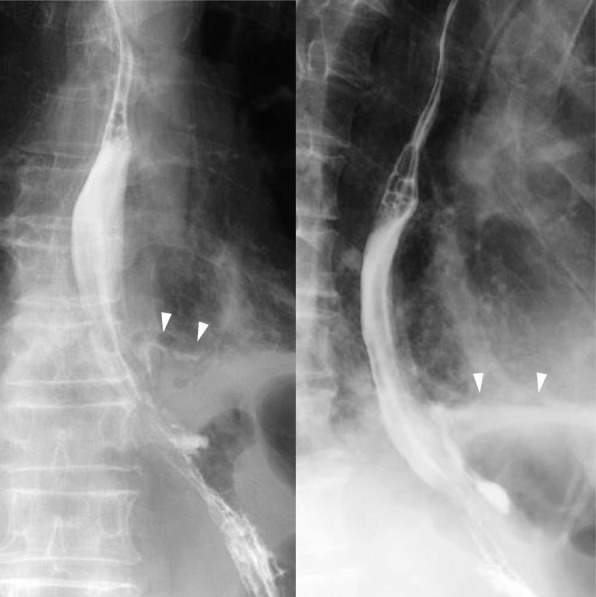
An esophagogram shows extravasation of contrast from the lower esophageal wall into the left thoracic cavity (arrowhead, extravasation of contrast)

A 66-year-old man with sore throat was referred to our hospital. He was diagnosed with stage IVA hypopharyngeal cancer, cT2N2bM0, based on the classification of the 8th edition of the Union for International Cancer Control. He underwent radical concurrent CRT that consisted of weekly cisplatin (30 mg/m^2^) and radiation (70 Gy/35fr) for larynx preservation (Fig. 1a). He received antiemetic therapy along with CRT to prevent chemotherapy-induced nausea and vomiting. Because of dysphagia and prolonged nausea due to the radiotherapy, a nasogastric tube was inserted on day 23 of CRT. Enteral nutrition was administered by intermittent injection three times a day, and the nasogastric tube was clamped overnight after the last daily administration of enteral nutrition. On the evening of day 27, the patient vomited, which was followed by severe left chest pain radiating to the back and the upper abdomen. During the vomiting episode, the nasogastric tube was unintentionally removed along with vomitus. A physical exam was negative for rebound tenderness in the abdomen; the symptoms were relieved by analgesics; and the patient was observed overnight. His symptoms returned in the morning, and his temperature was 38.2 °C. Laboratory testing showed an intense inflammatory response (CRP 8.02 mg/dL). Contrast-enhanced computed tomography (CT) revealed extensive mediastinal emphysema extending from the neck to the gastric cardia, a small amount of left pleural effusion, and no pneumothorax (Fig. 1b, c). Boerhaave syndrome was suspected at this point in the patient’s clinical course. Since his general status and vital signs were stable, he underwent esophagography to confirm the diagnosis and aid in treatment planning. Since reinsertion of a nasogastric tube was difficult, the esophagogram was obtained after the patient ingested contrast medium orally while being observed for aspiration. The esophagogram revealed extravasation of contrast from the lower esophageal wall into the left thoracic cavity (Fig. 2), which was diagnosed as an intrathoracic rupture type of Boerhaave syndrome.

**Fig. 3:**
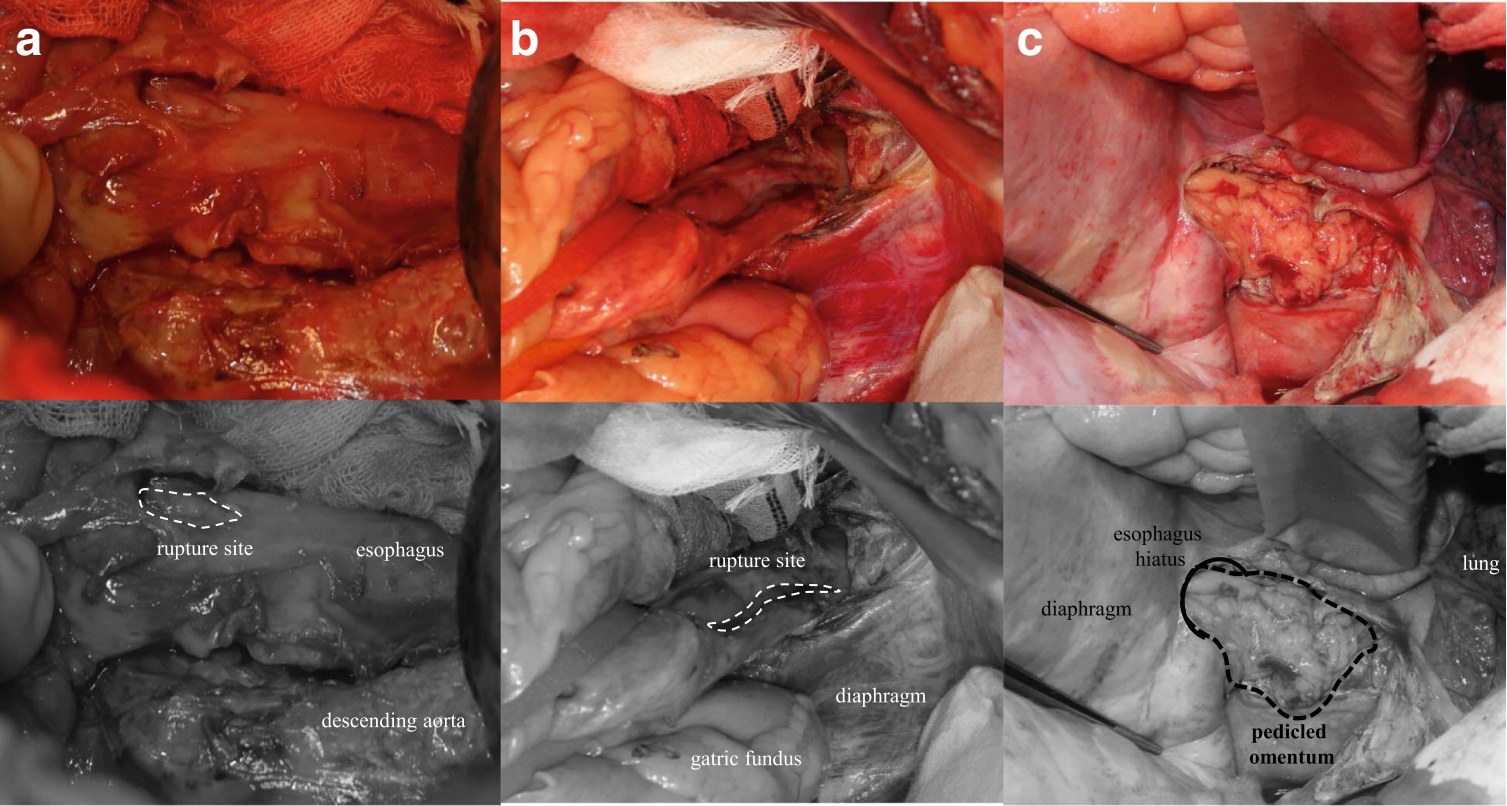
Surgical findings. **a** Via the thoracotomy incision, a rupture site is seen on the wall of the lower esophagus above the diaphragm. **b** Via a laparotomy midline incision in the upper abdomen, a 4-cm-long rupture is seen on the left side of the lower esophagus, and the rupture extends to the gastroesophageal junction. **c** Via the left thoracotomy, the ruptured esophageal wall was primarily repaired by closure with two-layer suturing and covered by pedicled omentum which was elevated thorough the esophageal hiatus (black dotted line, pedicled omentum)

He underwent emergency left thoracotomy in the right lateral decubitus position approximately 21 h from the onset. In preparation for left thoracotomy, surgery was first performed with the patient in the right half-lateral decubitus position. The left plural cavity contained contaminated pleural effusate, and a small rupture site was found in the left thoracic cavity. We dissected the lower lung ligament along the descending aorta, opened the mediastinal space for drainage, and identified the wall of the esophagus. Via the thoracotomy incision, we found a rupture site on the wall of the lower esophagus above the diaphragm (Fig. 3). The position of the patient was then changed for laparotomy by rotating the surgical bed. Subsequently, while exploring via a laparotomy midline incision in the upper abdomen, we found a 4-cm-long rupture site on the left side of the lower esophagus that extended to the gastroesophageal junction (Fig. 3b). The ruptured esophageal wall was primarily repaired by closure with two-layer suturing and covered by pedicled omentum that was elevated through the esophageal hiatus (Fig. 3c). A jejunostomy tube was placed for postoperative enteral nutrition, and the mediastinal and thoracic space were irrigated with saline and drained.

Immediately after the operation, the patient was placed on enteral nutrition therapy and a course of antibiotics. The drain was changed for the remaining mediastinal abscess on postoperative day (POD) 10; otherwise, the postoperative course was uneventful. On POD 16, the patient was transferred to head and neck surgery to finish CRT and was discharged on POD 56. He has survived without relapse for 10 months after surgery.

### Discussion

Spontaneous esophageal rupture, also known as Boerhaave syndrome, is a very serious life-threatening benign disease of the gastrointestinal tract. The rupture is caused by a sudden increase in intraesophageal pressure, leading to a full-thickness transmural rupture of the normal esophagus. Vomiting is the most frequent cause of Boerhaave syndrome; 70% of patients are reported to have developed Boerhaave syndrome from vomiting after heavy eating and drinking [1]. However, Boerhaave syndrome has been reported in various patients, including those with gastrointestinal stenosis, ileus, vomiting during pretreatment for an endoscopic examination, and vomiting after general anesthesia [4–6]. Thus, Boerhaave syndrome can occur irrespective of the cause of vomiting. The signs and symptoms of Boerhaave syndrome that are typically seen after heavy alcohol use occur in a sequence of events that include severe vomiting, mild hematemesis, and substernal chest pain [1]. Left pleuritic chest pain may radiate to the epigastrium, substernal area, or back. This characteristic medical history is the key to the diagnosis of Boerhaave syndrome, which should be the first suspected possible diagnosis. Moreover, imaging is very important for the diagnosis. The typical findings of chest radiography and CT are subcutaneous emphysema, mediastinal emphysema, and pleural effusion [1]. A definitive diagnosis can be obtained by esophagography, which aids in identifying the site and type of rupture, which include intrathoracic and rupture localized to the mediastinum. Studies have reported that 84% of ruptures were located in the left wall of the lower third of the esophagus, a site that is anatomically vulnerable [7]. Based on a patient’s general condition and type of esophageal rupture, the treatment approach can be either surgical or conservative. Surgical treatment is commonly used for intrathoracic rupture [8]. The principle of surgical treatment is primary suture repair of the rupture and adequate drainage of the mediastinum and thoracic cavity [9]. If the esophagus is seriously damaged by severe mediastinitis so that a suture repair cannot be performed, placement of a T-tube, oversewing of adjacent tissue such as a pedicled omentum, or esophagectomy that includes the rupture site and second-stage reconstruction, are often performed [7]. Conservative treatment has been reported for patients with mild symptoms, without severe infection, and with the rupture localized to the mediastinal space, with the esophagus completely draining through the rupture site [10]. If after conservative treatment the patient’s condition deteriorates, surgical treatment must be performed immediately. Delayed diagnosis and treatment can rapidly lead to severe life-threatening infections such as empyema and mediastinitis, and multiple organ failure. Therefore, especially in patients who are undergoing CRT for tumors, the rapid diagnosis and appropriate treatment of Boerhaave syndrome are extremely important for avoiding sepsis and a lethal outcome [1, 7]. Although the mortality rates of patients with Boerhaave syndrome previously ranged from 30 to 40%, improvements in the postoperative management of patients treated for Boerhaave syndrome, which include the administration of nutritional therapy, have recently led to better outcomes, with mortality rates ranging from 3.7 to 7.9% [1, 7, 11].

With the inclusion of our case, three cases of Boerhaave syndrome during CRT for hypopharyngeal cancer have been reported (Table 1) [6, 12]. In all three cases, chemotherapy-induced vomiting triggered Boerhaave syndrome. In our case, the causes of Boerhaave syndrome are thought to include not only chemotherapy-induced vomiting but also pharyngeal stenosis associated with the tumor, radiotherapy-induced edema, and relaxation failure. That is, elevated pressure due to vomiting was directly focused on the lower esophagus, leading to esophageal rupture. Kiyuna et al. reported that CRT for pharyngeal cancer can lead to serious problems such as dysphagia. The pathogenesis of dysphagia has been reported to include mucositis, pharyngeal edema, stenosis due to scarring, attenuation of pharyngeal contraction due to fibrosis, relaxation failure, and delayed reflexes [3].

**Table 1:** Previously reported cases of Boerhaave syndrome during CRT

No.	Author	Year	Age	Sex	Days from CRT to onset	Rupture site	Rupture type	Hours from onset to treatment	Treatment	Outcome
1	Okumura	2014	61	M	19	Left side of the lower esophagus	Intrathoracic	19	Surgical (primary suture and covered by omentum)	Postoperative death (24 POD)
2	Furukawa	2015	58	M	8	Left side of the lower esophagus	Mediastinal	8	Conservative (endoscopic stent replacement)	Survive (12 months)
3	Our case	2017	66	M	28	Left side of the lower esophagus	Intrathoracic	21	Surgical (primary suture and covered by omentum)	Survive (11 months)

Regarding the two other reported cases, although the patient reported by Okumura et al. had hypopharyngeal cancer without stenosis, chronic, severe radiotherapy-induced mucositis in addition to vomiting was associated with the development of Boerhaave syndrome. Furukawa et al. reported that the effect of radiotherapy on their patient with Boerhaave syndrome was unclear; it occurred in association with circumferential esophageal cancer in the mid thorax instead of in association with hypopharyngeal cancer.

In all three cases, treatment was initiated within 24 h after onset. However, Okumura et al. reported that the general condition of their patient worsened because of failed suture repair, and the patient died after surgery. The authors thought that the patient was in poor overall condition because of the tumor and CRT, and was also immunocompromised with protracted wound healing. Kiyuna et al. reported that patients with a tumor who undergo CRT often have poor overall condition because of immunosuppression and protracted wound healing [3]. Such patients who develop Boerhaave syndrome are probably at greater risk of dying than patients with normal immune status. The initiation of enteral nutrition during the early postoperative period is recommended, especially for patients treated for conditions such as Boerhaave syndrome that manifest as acute abdomen, because enteral nutrition maintains the functions of the immune system in the intestinal tract and prevents atrophy of the intestinal mucosa [13]. Moreover, the rate of postoperative infectious complications in patients undergoing enteral nutrition is lower than that of patients undergoing intravenous nutrition [14]. Therefore, enteral nutrition therapy for our patient was started immediately after surgery to prevent further decline in his immune status.

Physicians should be aware of Boerhaave syndrome when caring for patients with head and neck cancer who may have pharyngeal stenosis due to the tumor or radiotherapy, and who undergo chemotherapy that might cause vomiting. In addition, since patients undergoing CRT are often in poor overall condition, Boerhaave syndrome must be diagnosed early and treated promptly.

## Conclusions

We encountered a patient who developed Boerhaave syndrome during CRT for hypopharyngeal cancer, and we successfully diagnosed the syndrome and performed surgery during the early stage of the syndrome. Patients with head and neck cancer are at risk for developing Boerhaave syndrome during CRT, because of the combination of hypopharyngeal cancer with stenosis and CRT, which leads to chemotherapy-induced vomiting, radiotherapy-induced edema, relaxation failure, and delayed reflexes. Therefore, awareness of this condition is important so that appropriate treatment can rapidly be implemented to increase the likelihood of a good outcome.

## Abbreviations

CRT: Chemoradiotherapy
CT: Computed tomography
POD: Postoperative day
